# Skin symptoms and their impact on quality-of-life in adults with osteogenesis imperfecta

**DOI:** 10.1210/jendso/bvag182

**Published:** 2026-09-03

**Authors:** Winnie Liu, Jonathan Xu, Apoorva Salvi, Suephy Chen, Lars Folkestad, Jorg Oliver Semler, Eric Orwoll

**Affiliations:** Department of Medicine, Division of Endocrinology, Diabetes & Clinical Nutrition, Oregon Health & Science University, Portland, OR 97239, USA; Department of Medicine, Division of Endocrinology, Diabetes & Clinical Nutrition, Oregon Health & Science University, Portland, OR 97239, USA; Department of Medicine, Division of Endocrinology, Diabetes & Clinical Nutrition, Oregon Health & Science University, Portland, OR 97239, USA; Department of Dermatology, Duke University School of Medicine and Durham VA Medical Center, Durham, NC 27710, USA; Department of Endocrinology, Odense University Hospital, 5000 Odense C, Denmark; Department of Molecular Endocrinology, Odense University Hospital, 5000 Odense C, Denmark; Department of Clinical Research, University of Southern Denmark, 5230 Odense, Denmark; Department of Pediatrics, Faculty of Medicine and University Hospital Cologne, University of Cologne, 50937 Cologne, Germany; Department of Medicine, Division of Endocrinology, Diabetes & Clinical Nutrition, Oregon Health & Science University, Portland, OR 97239, USA

**Keywords:** skin, type 1 collagen, osteogenesis imperfecta, brittle bone disease, extracellular matrix

## Abstract

Osteogenesis imperfecta (OI) is a genetic connective tissue disorder caused by abnormalities in type I collagen, which is abundant in skin. Yet, data on dermatologic manifestations and their impact on quality-of-life (QOL) are limited. This study evaluated the prevalence of skin symptoms in adults with OI compared with adults without OI and assessed their effect on QOL. In this cross-sectional, survey-based study, adults with OI were recruited via US and European OI foundations, and comparators were recruited from an Oregon Health & Science University research registry. Participants completed a 20-item skin symptom questionnaire; those reporting bothersome or itchy skin also completed validated QOL instruments (Skindex-29 and ItchyQoL). Group and severity-stratified comparisons were analyzed using t-tests, chi-square tests, and analysis of variance (*P* < .05). A total of 821 surveys were analyzed (547 OI). Adults with OI reported significantly more skin symptoms than comparators, most commonly easy bruising (78%), dry skin (72%), and excessive sweating (66%). Women with OI reported more symptoms than men. Queries about QOL revealed that over half of OI participants reported bothersome skin, with higher Skindex-29 and ItchyQoL scores than comparators. Symptom prevalence also varied by OI severity: severe OI was associated with pruritus and rashes, while mild OI was associated with bruising and fragile nails. Skin symptoms are a common and clinically meaningful extra-skeletal manifestation of OI. Differences in skin symptoms may be influenced by sex, age, and OI subtype, highlighting the need for further studies as recognition of these dermatologic manifestations may guide targeted management.

Osteogenesis imperfecta (OI) is a heritable connective tissue disease primarily caused by mutations in a variety of genes that result in defects in the synthesis of type I collagen. Despite the disorder's genetic heterogeneity, 85% of OI cases result from dominantly inherited mutations in the *COL1A1* and *COL1A2* genes, which encode the α-polypeptide chains of type I collagen [1]. The remaining 15% represent diverse genetic etiologies stemming from noncollagen genes. These less common OI types (types V–XXII) implicate genes involved in bone mineralization, osteoblast differentiation, and the post-translational modification, folding, and cross-linking of collagen molecules [2].

The phenotypic presentations of OI are broad, with clinical features ranging from mild skeletal fragility to perinatal lethality. The clinical classification of OI follows the Sillence system, which outlines five primary classes (types I–V) that encompass most of the OI population. Type I (nondeforming with blue sclerae) is the mildest form with increased fracture risk and minimal deformity; type II (perinatally lethal) is the most severe, with in utero fractures and death in the neonatal period; type III (progressively deforming) is the most severe form of OI in individuals surviving the neonatal period. Type IV (common variable with normal sclerae) presents with intermediate severity; and type V features unique calcification of interosseous membranes [3].

Although OI is characterized by bone fragility, the qualitative and quantitative collagen abnormalities in OI are systemic and manifest in other collagen-containing tissues such as the respiratory, cardiovascular, and integumentary systems [4]. Collagen is the most abundant protein in the body and plays a key role in the structure and function of the extracellular matrix (ECM). In skin, type 1 collagen accounts for 85% of the dermal ECM and provides strength, structural support, and elasticity [5].

Small studies have demonstrated structural and functional skin abnormalities in OI, including reduced dermal collagen content, altered collagen-to-elastic fiber ratios, disorganized collagen fibrils with variable diameter, and reduced elasticity with increased distensibility on mechanical testing. Survey-based studies and case reports document increased skin symptoms in OI patients, including fragile skin, delayed wound healing, easy bruising, and overlap syndromes with Ehlers-Danlos and elastosis perforans serpiginosa [6‐10]. While the existing literature suggests abnormalities in skin structure, function in individuals with OI, the clinical scope and prevalence of skin symptoms and their impact on quality-of-life (QOL) have not been adequately explored.

## Purpose

This study aimed to assess the prevalence of common skin symptoms and their impact on QOL in individuals with OI using questionnaire-based symptomatic, emotional, and functional indices. We hypothesized that skin symptoms are more prevalent in individuals with OI compared to comparators and that these symptoms significantly impair QOL.

## Methods

### Study participants and survey design

This cross-sectional study examined dermatologic symptoms in adults with OI compared with controls and differences between OI types. A suite of surveys was developed and distributed to volunteers from two cohorts: (1) individuals with OI who were participants in patient registries created by the Osteogenesis Imperfecta Foundation and Osteogenesis Imperfecta Federation Europe; and (2) participants from the Research Volunteer Registry and Biorepository—a registry of healthy volunteers at OHSU who are interested in and consented to be contact about research. These participants were recruited through health fairs and community events; direct advertisements (OHSU Research Opportunities webpage, flyers, brochures) and referrals from other OHSU IRB-approved studies. Participants were between 18 and 89 years, generally healthy, willing to be contacted periodically to update their information, and able to provide consent. We excluded participants with active infectious disease (eg, HIV, hepatitis), serious medical condition or medications that would interfere with their ability to participate (such as active cardiac or pulmonary disease, diabetes mellitus, clinically significant cancer diagnosis, active infectious disease, and active inflammatory diseases).

The survey suite consisted of four questionnaires that were presented in the following sequence. First, we administered a questionnaire that included general demographic items, such as age, sex, and race/ethnicity. Respondents with OI were asked to report their perceived OI severity as mild, moderate, severe or known/prefer not to answer. Second, we administered a questionnaire of skin symptoms (The General Skin Symptoms Questionnaire [GSSQ]). Finally, if respondents answered “yes” to the symptom question “Does your skin bother you?”, respondents were prompted to complete the Skindex-29 questionnaire [11]. If respondents answered “yes” to the symptom question “Does your skin itch?”, they were prompted to complete ItchyQoL [12].

The GSSQ is a 20-item, yes/no assessment of skin symptoms (Table S1) [13]. The GSSQ was developed collaboratively by the authors, including physicians who treat patients with OI, with input from persons with OI and their advocates. Skindex-29 and ItchyQoL are clinically validated questionnaires used to quantitatively measure the burden of skin disease on QOL [11, 12]. Skindex-29 is a 30-question survey that characterizes the effects of skin disease by dividing its impact on QOL into symptomatic (eg, “My skin hurts”), emotional (eg, “My skin condition makes me feel depressed”), and functioning (eg, “My skin condition affects my social life”) categories. There is also a Skindex-29 composite score that includes the three QOL domains. It provides scaled scores for each domain, ranging from 0 to 100, with 0 representing no effect and 100 representing constant affliction [11]. ItchyQoL is a 22-item questionnaire that includes the same three QOL categories but instead measures both frequency and severity on a pruritus-specific basis (eg, “My skin hurts because of my itchy skin condition”). Similar to Skindex-29, scoring of ItchyQoL consists of three domain scale scores (symptomatic, emotional, and functional) from of 1-5, with 1 representing a frequency of never and a severity of not bothered, and 5 representing a frequency of “all the time” and a severity of “severely bothered” [12].

Survey data were securely collected using REDCap (Research Electronic Capture) electronic data capture tools [14, 15]. The study was approved by the Institutional Review Board at OHSU and all participants provided informed consent. The survey was open from September 2024 to December 2024.

### Statistical analyses

The primary outcome measures were responses to the skin symptoms survey, expressed as the number of participants in the comparator and OI groups stratified by sex and OI severity who answered “Yes” to each question, and the domain scale scores from Skindex-29 and ItchyQoL. To assess significant differences in responses to the skin symptom survey, independent samples t-tests and chi-squared tests of independence were utilized to compare comparator and total OI cohorts, and across OI severity subgroups, respectively. To examine Skindex-29 and ItchyQoL domain scale scores stratified by OI severity, one-way analysis of variance was used. Primary analyses focused on prespecified, hypothesis-driven comparisons using validated QOL instruments. Since questionnaire subscales represent conceptually related and correlated domains rather than independent endpoints, strict correction for multiple testing was not performed, and secondary and subgroup analyses were interpreted cautiously.

All analyses were performed at OHSU using Stata (StataCorp LLC, USA) version 18 software, with *P*-values <.05 considered statistically significant.

## Results

Survey invitations were emailed to 985 individuals from the Research Volunteers Repository contact list and 274 (27.8%) of them responded and completed the surveys. Five hundred and eighty nine individuals with OI responded and completed the surveys. We cannot calculate a response rate since the survey link was distributed through email lists, websites, and social media posts. Of the 589 people with OI who initially responded, 42 were excluded due to reporting an age of less than 18 years. Ultimately, 821 (66.6% OI respondents) surveys were analyzed. The mean age was similar between respondents with OI and the comparator group (49.1 ± 15.8 vs 50.6 ± 16.9 years, respectively, *P* = .2). Most respondents were female (77.1% in the OI group and 74.5% in the comparator group, *P* = .52) and both groups were predominantly White (>83%). There was a statistically significant difference in the racial distribution, with slightly more Hispanic/Latino individuals in the OI group. Among the 547 respondents with OI, 302 (55.2%) described their disease severity as mild, 179 (32.7%) as moderate, and 46 (8.4%) as severe (Table 1).

**Table 1:** Demographic and clinical characteristics of OI and comparator survey respondents

Characteristics	OI (N = 547)	Comparator (N = 274)	*P*-value
Age, mean (SD), years	49.12 (15.79)	50.60 (16.88)	.217
Age group			.011
18-40 years	178 (32.5)	96 (35.0)	
41-60 years	225 (41.1)	85 (31.0)	
≥61 years	144 (26.3)	93 (33.9)	
Sex assigned at birth			.518
Female	422 (77.1)	204 (74.5)	
Male	121 (22.1)	69 (25.2)	
Prefer not to answer	4 (0.7)	1 (0.4)	
Race/ethnicity			.017
White	483 (88.3)	230 (83.9)	
Asian	13 (2.4)	19 (6.9)	
Hispanic/Latino	29 (5.3)	9 (3.3)	
Black or African American	6 (1.1)	2 (0.7)	
American Indian or Alaska Native	1 (0.2)	1 (0.4)	
Native Hawaiian or Other Pacific Islander	1 (0.2)	0 (0.0)	
Other/Prefer not to answer	14 (2.6)	13 (4.7)	
Self-reported OI severity			
Mild	302 (55.2)	—	
Moderate	179 (32.7)	—	
Severe	46 (8.4)	—	
Prefer not to answer/Not sure	20 (3.7)	—	

### General skin symptoms questionnaire

#### OI vs comparator

**Figure 1:**
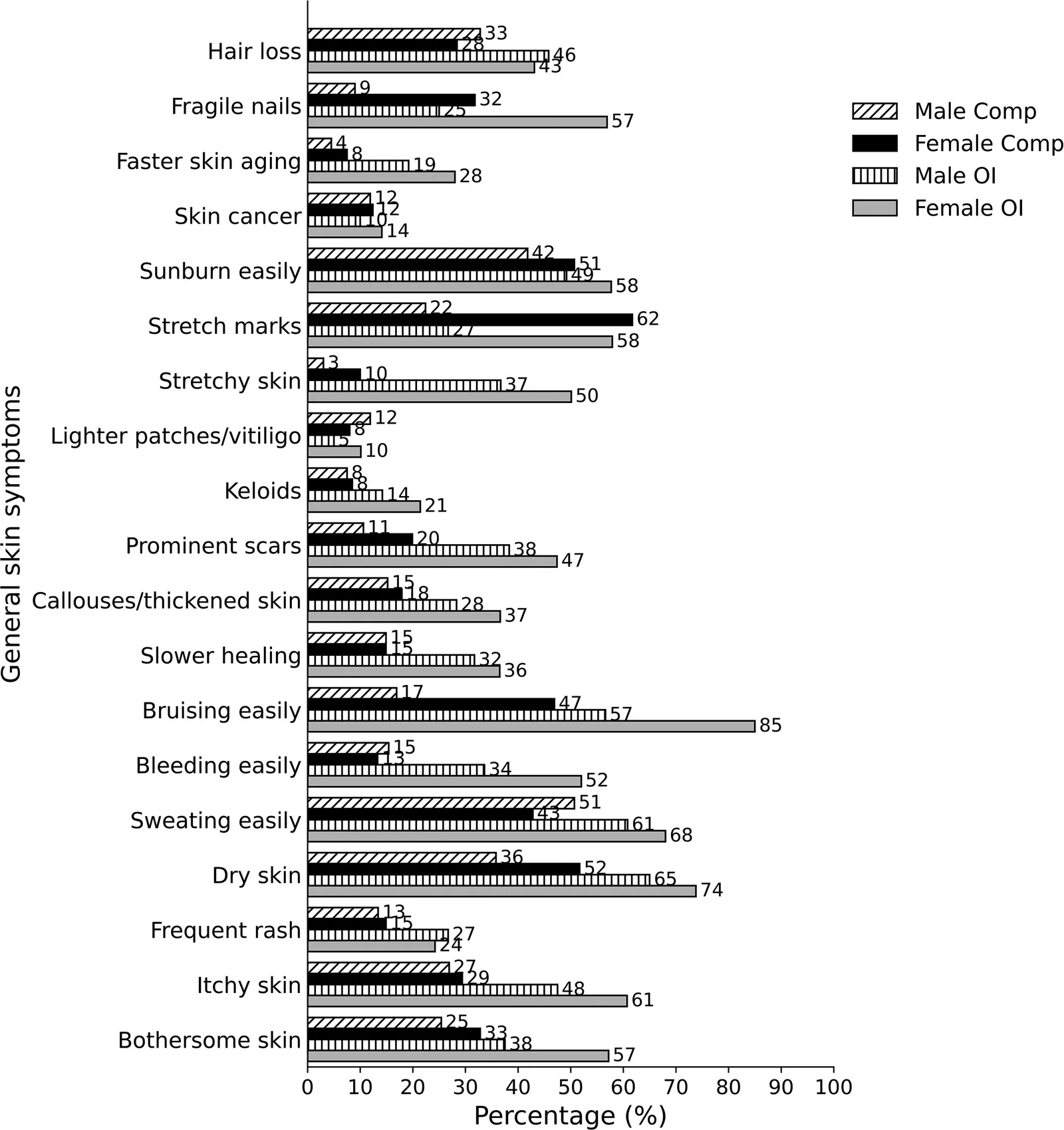
Prevalence of general skin symptoms in individuals with osteogenesis imperfecta compared with a comparator group, by sex.

Survey respondents with OI reported a substantially higher prevalence of skin symptoms than comparators. Statistically significant differences were observed with nearly all skin-related questions except those regarding skin cancer, sunburns, vitiligo, and stretch marks. The most common symptoms in OI were easy bruising (78.3%), dry skin (72.0%), and excessive sweating (66.2%). The largest between-group differences were seen for easy bruising (39%) and stretchy skin (39%), (Fig. 1).

#### Sex-stratified analyses

Women with OI reported more skin symptoms than women in the comparator group in 15 out of the 20 items in the GSSQ. The same pattern was observed for males with OI, who reported more symptoms on 11 of the 20 items (Fig. 1 and Table S1) [13].

#### Female OI vs male OI

Within the OI cohorts, females reported significantly more skin symptoms than males, with the largest differences observed for fragile nails (31.9%), stretch marks (31.2%), and easy bruising (28.4%). Overall, this pattern of sex differences in skin symptoms was similar to that of the comparator population (Fig. 1).

#### Mild, moderate, and severe OI

**Figure 2:**
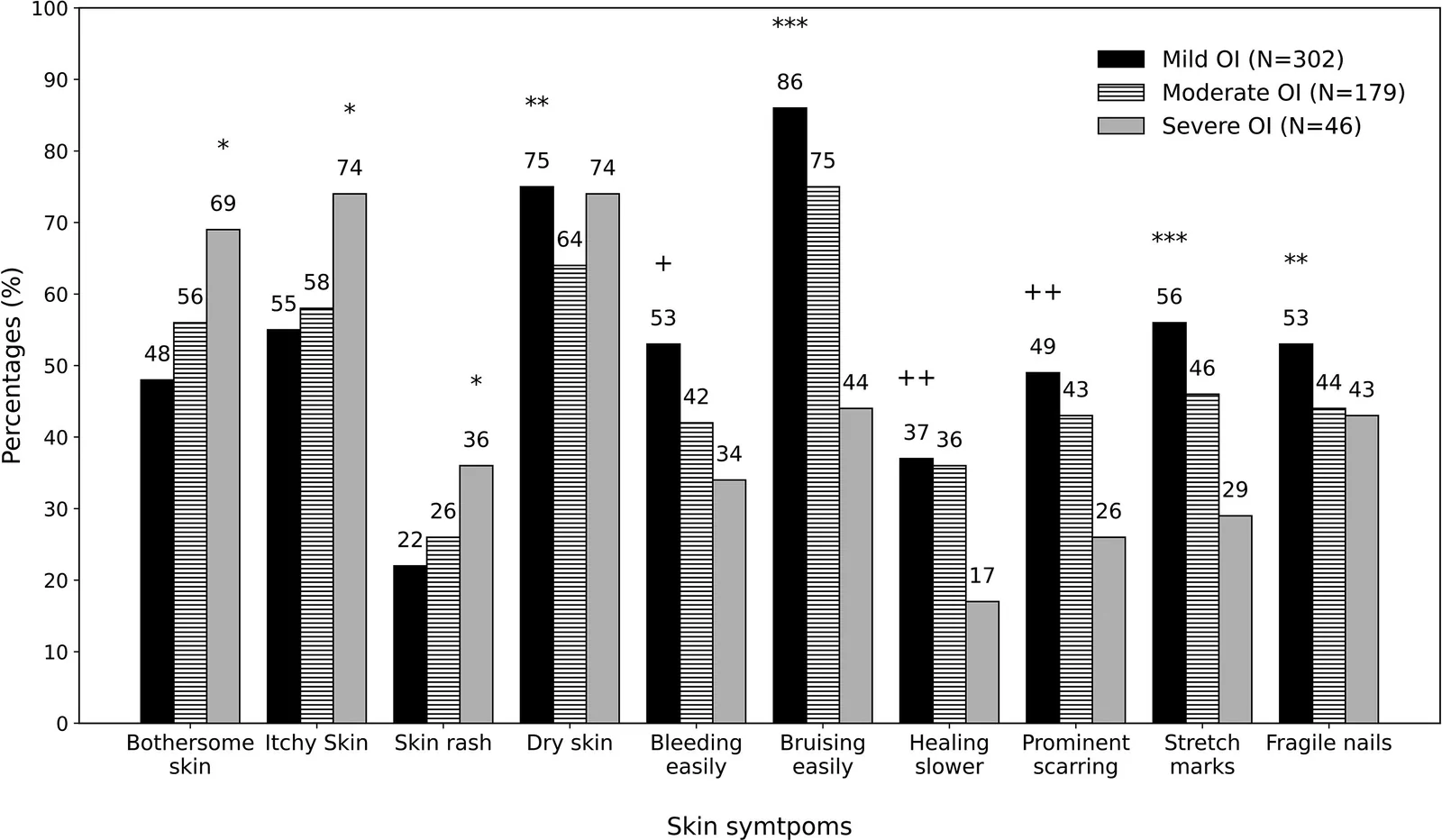
Prevalence of skin symptoms by osteogenesis Imperfecta severity. Signifies statistically significant difference between: *mild vs severe. **mild vs moderate. ***mild vs moderate, milder vs severe, and moderate vs severe. +mild vs moderate and mild vs severe. ++mild vs severe and moderate vs severe.

Individuals with mild, moderate, and severe OI answered similarly to 10 out of 20 skin questions. A higher proportion of those with severe OI than those with mild OI reported bothersome skin, itchy skin, and frequent rashes. On the other hand, a group of symptoms was significantly more common among those with milder OI, including bruising, bleeding, slow healing, scarring, stretch marks, and brittle nails (Fig. 2).

### Skindex-29

#### OI vs comparator

Over half of participants (53%) with OI reported bothersome skin and completed the Skindex-29, compared with 31% of comparators. Among those with bothersome skin, individuals with OI had significantly higher Skindex-29 symptomatic scores than comparators (40.1 ± 18.5 vs 31.5 ± 19.0; *P* < .001); nevertheless, both mean scores fall within the severe category and there were no differences in functional or emotional scores. The composite scores were also higher in OI respondents than in comparators (30.0 ± 17.6 vs 25.0 ± 15.1, respectively; *P* = .02).

#### Sex-stratified analyses

Men with OI had significantly higher symptomatic scores (34.0 ± 17.8 vs 20.8 ± 16.9, *P* = .011) and composite scores (24.6 ± 17.1 vs 15.7 ± 10.3, *P* = .049) than male comparators, whereas emotional and functional subscales did not differ. Women with OI had higher symptomatic scores than comparators (41.3 ± 18.5 vs 34.3 ± 18.7, *P* = .008). Women with and without OI responded similarly for emotional, functional, and composite scores (Table 2).

**Table 2:** Skindex-29 and ItchyQoL subscale and composite scores in individuals with osteogenesis imperfecta compared with a comparator group, overall and by sex

Skindex-29 subscale and composite scores, mean (SD)	Overall			Female			Male			OI Female vs Male
	OI	Comparator	*P*-value	OI	Comparator	*P*-value	OI	Comparator	*P*-value	*P*-value
Emotional, n = 343	32.5 (21.95)	30.2 (20.71)	.399	33.7 (21.96)	33.0 (21.64)	.821	26.1 (21.24)	19.6 (12.13)	.235	.**038**
Symptoms n = 342	40.1 (18.53)	31.5 (19.04)	**<**.**001**	41.3 (18.51)	34.3 (18.68)	.**008**	34.0 (17.77)	20.8 (16.91)	.**011**	.**018**
Functional, n = 344	17.1 (18.81)	13.2 (14.71)	.083	17.8 (19.31)	14.9 (15.52)	.265	13.7 (16.36)	6.7 (8.73)	.103	.188
Composite, n = 353	30.0 (17.63)	25.0 (15.12)	.**020**	31.1 (17.62)	27.4 (15.30)	.131	24.6 (17.11)	15.7 (10.26)	.**049**	.**028**

ItchyQoL subscale scores Mean (SD)	Overall			Female			Male			OI Female vs Male
	OI Group	Comparator	*P*-value	OI	Comparator	*P*-value	OI	Comparator	*P*-value	*P*-value
Symptoms, n = 338	1.5 (0.74)	1.2 (0.81)	.**002**	2.5 (0.74)	2.2 (0.84)	.**017**	2.3 (0.76)	2.0 (0.71)	.114	.075
Functional, n = 336	1.1 (0.83)	0.9 (0.79)	.072	2.2 (0.84)	2.0 (0.85)	.337	1.8 (0.76)	1.5 (0.40)	.088	.**008**
Emotional, n = 348	1.0 (0.94)	0.9 (0.91)	.368	2.1 (0.96)	2.0 (0.98)	.751	1.8 (0.86)	1.6 (0.58)	.297	.099

Significant *P* values appear in bold.

Comparing sexes within OI, women scored significantly higher on the emotional subscale (33.7 ± 22.0 vs 26.1 ± 21.2, *P* = .038), symptomatic subscale (41.3 ± 18.5 vs 34.01 ± 17.8, *P* = .018), and composite scores (31.1 ± 17.6 vs 24.6 ± 17.1, *P* = .008) than men (Table 2).

#### Mild, moderate, and severe OI

There were no significant differences among mild, moderate, and severe OI respondents in any of the Skindex-29 composite and subscale scores.

### ItchyQoL

#### OI vs comparator

More than half (58%) of participants with OI reported itchy skin and completed the ItchyQoL instrument, compared to a much smaller proportion of the reference participants (28.6%) (Table 2). The OI group had a statistically significantly higher symptomatic score than the reference group (1.48 ± 0.74 vs 1.17 ± 0.81, *P* = .003). There were no significant score differences between groups in either the functional or emotional subscale scores or in the overall frequency categories for these domains (Table 2).

#### Sex-stratified analyses

Among men, participants with OI and comparators scored similarly (Table 2). In women, a higher symptomatic score was observed in OI group (2.52 ± 0.74 vs 2.24 ± 0.84, *P* = .017) while functional and emotional subscales were similar between OI and comparators.

Women with OI reported a higher itch-related burden than men with OI, with a higher functional subscale score (2.16 vs 1.82, *P* = .008), but similar QOL burden in the symptoms and emotional domains (Table 2).

#### Mild, moderate, and severe OI

ItchyQoL scores did not differ by self-reported OI severity (mild, moderate, or severe).

## Discussion

Adults with OI reported substantially higher frequencies of skin symptoms than did the comparator population. Among OI respondents who experienced bothersome skin symptoms, we observed a negative impact on QOL. Collectively, these findings indicate that skin symptoms are common and clinically meaningful in adults with OI and contribute to reduced QOL.

A previous survey-based study of 959 adults with OI examined a range of extra-skeletal manifestations and reported a higher prevalence of dry skin compared with national benchmark datasets, including the National Health Interview Survey [7]. The study assessed eight skin-related items and found that skin symptoms were associated with adverse effects on QOL. A second study analyzing medical records from 568 individuals with OI (including 282 adults) reported skin abnormalities in 15% of participants, categorized as morphological abnormalities (4.2%), cutis laxa (11.6%), and skin lesions (1.1%) [8].

In comparison, our survey included a broader set of specific skin-related symptoms, allowing a more comprehensive characterization of dermatologic manifestations in OI. In addition, we included a comparator cohort with similar characteristics who were presented with the same survey questions. This allowed us to conclude that men and women with OI have higher prevalences of dermatologic symptoms. We similarly observed a negative impact of skin symptoms on QOL, with a greater symptom burden and a particularly pronounced QOL effects among women with OI. This is consistent with trends observed in the general population and likely reflect sex-related differences in skin structure and physiology, the effects of sex hormones, and sociocultural and environmental factors [16]. Taken together, these findings reinforce the importance of recognizing skin manifestations as a clinically meaningful component of the extra-skeletal phenotype of OI.

We found that the prevalence of several skin symptoms differed by self-reported OI severity. One potential explanation may be the distinct effects of qualitative vs quantitative defects in type I collagen on skin structure and function. Qualitative collagen abnormalities, which are more typical of severe OI, may disrupt collagen fibril organization and structure. This altered dermal architecture may promote local tissue stress and inflammation, contributing to skin manifestations such as rashes or irritation. The explanation may be further supported by documented cases of Elastosis perforans serpiginosa, which is an inflammatory response to abnormal elastic fibers and are eliminated through the epidermis in patients with OI [9, 17]. In contrast, quantitative collagen defects, more characteristic of milder OI, may lead to reduced skin thickness and increased capillary fragility, resulting in symptoms such as easy bruising and bleeding [14]. Differences in symptom reporting may also reflect variation in symptom perception across OI severity levels. Individuals with more severe OI more frequently reported symptoms that were physically noxious or visually apparent, such as rashes or pruritic lesions. Conversely, respondents with milder OI more often experienced symptoms associated with lower levels of irritation, including easy bruising, dry skin, and fragile nails. It is possible that individuals with severe OI experience multiple concurrent sources of physical discomfort, such as chronic musculoskeletal pain, which may alter the perceived burden or reporting of skin-related symptoms. While no conclusions can be drawn directly from these results, it lays the groundwork for future genotype–phenotype studies.

This study has several strengths. We sampled a large and diverse group of adults with OI, spanning the various levels of OI severity. We used a detailed questionnaire to assess a range of skin symptoms and validated patient-reported outcome measures to evaluate the effect of skin symptoms on QOL (ItchyQoL and Skindex-29). These instruments enabled quantitative assessment of both symptom burden and QOL impact. Compared with prior reports, this study offers a comprehensive and focused evaluation of skin symptoms specifically in adults with OI, a population that may experience dermatologic manifestations distinct from those observed in children. A major strength is the inclusion of a simultaneously studied comparator population, allowing contextual interpretation of symptom frequency and impact. Nonetheless, several limitations warrant consideration. The use of a convenience sample from the OI community introduces the potential for selection and response bias, and reliance on self-reported data may be subject to recall bias and subjective interpretation. The use of a convenience sample from the OI community introduces the potential for response bias, and reliance on self-reported data may be subject to recall bias and subjective interpretation. The absence of a formal evaluation of clinical OI type and genotyping precluded genotype–phenotype correlation analyses to determine whether specific collagen mutations differentially affect cutaneous manifestations.

Our sample did not allow us to consider possible racial or cultural differences in skin involvement in OI, and we lacked information about medical comorbidities that may have influenced the prevalence or impact of skin symptoms.

Overall, our findings support skin involvement as a clinically meaningful extra-skeletal manifestation of OI. Although no definite conclusions can be drawn regarding the biological basis for these observations, it may suggest the central role of type I collagen—the primary protein affected in OI—in maintaining dermal structure and function. Abnormal collagen quantity or quality can compromise dermal integrity, elasticity, and wound healing [6, 18], providing a mechanistic explanation for the increased prevalence of skin fragility, bruising, and dryness observed in this and other studies. Further research, including mechanistic studies of ECM biology and detailed clinical characterization of cutaneous phenotypes in OI, is needed to elucidate the underlying pathways of skin involvement. The impact of skin abnormalities on other medical issues (eg, surgical care) warrants more attention. Improved clinical recognition of these manifestations may inform targeted management strategies and ultimately enhance QOL for individuals living with OI.

## Data Availability

Some or all datasets generated during and/or analyzed during the current study are not publicly available but are available from the corresponding author on reasonable request.
